# Association of Dietary Sodium-to-Potassium Ratio with Cardiometabolic Risk Factors in Korean Adults: Findings from the Korean National Health and Nutrition Examination Survey

**DOI:** 10.3390/nu15143134

**Published:** 2023-07-13

**Authors:** Seo-Young Baek, Hye-Kyeong Kim

**Affiliations:** Department of Food Science and Nutrition, The Catholic University of Korea, Bucheon 14662, Republic of Korea; seoyoung9933@naver.com

**Keywords:** sodium-to-potassium ratio, hypertension, cardiometabolic risk, metabolic syndrome, food group

## Abstract

High-sodium and low-potassium intakes are interdependently linked to hypertension and cardiovascular diseases. We investigated the associations of dietary sodium-to-potassium (Na/K) ratio with cardiometabolic risk factors in 12,996 Korean adults (≥30 years) from the Korean National Health and Nutrition Examination Survey Ⅶ (2016–2018). Food intake was assessed through 24 h dietary recall data. Participants were divided into thirds based on their dietary Na/K ratio, with mean molar Na/K ratios of 1.11 (low), 1.92 (medium), and 3.21 (high). Although no significant associations were found between the dietary Na/K level and the risk of hypertension, obesity, and diabetes in all participants, the high Na/K ratio group had a higher risk of hypertension compared to the low Na/K ratio group in older adults (≥65 years) after adjusting for confounding factors (odds ratio = 1.38, 95% confidence interval: 1.10–1.72). Moreover, a higher Na/K ratio was associated with an increased risk of metabolic syndrome (MetS) in all participants (*p* for trend = 0.0020). Within MetS components, abdominal obesity, elevated triglycerides, and elevated blood pressure were positively associated with the Na/K level. The food groups positively associated with a lower Na/K ratio were fruits, unsalted vegetables, nuts, potatoes, and dairy products. These findings suggest that a high dietary Na/K ratio may be an important risk factor for hypertension in older adults and MetS in all adults.

## 1. Introduction

In the past few decades, there has been a global increase in the incidence of chronic diseases, such as obesity, hypertension, and diabetes [1,2,3]. These diseases are well-known risk factors for cardiovascular diseases (CVD), the leading causes of death and disability worldwide [4]. Additionally, metabolic syndrome (MetS), a group of interrelated risk factors, has become increasingly prominent in recent years, and its prevalence and impact on public health are expected to continue to increase [5,6]. These risk factors include abdominal obesity, insulin resistance, dyslipidemia, and elevated blood pressure (BP). Substantial evidence shows that MetS is associated with an increased risk of cardiometabolic diseases, such as coronary heart disease, stroke, and type 2 diabetes [7,8].

As an important modifiable lifestyle factor implicated in cardiometabolic diseases, diet has attracted the attention of investigators. Previous studies have demonstrated that higher sodium and lower potassium intakes were associated with higher BP [9,10], and a high potassium intake was associated with a lower BP by mitigating the effect of high sodium intake on BP levels [11]. Therefore, restricting sodium intake and increasing potassium intake have been widely recommended as a strategy to lower BP and CVD risk because hypertension is a primary risk factor for CVD [12]. The dietary reference intake of sodium is less than 2300 mg/day for Koreans, based on chronic disease risk reduction (CDRR), and the adequate intake (AI) of sodium and potassium is set at 1500 and 3500 mg/day, respectively [13]. Meanwhile, several studies have reported that the dietary sodium-to-potassium (Na/K) ratio appears more associated with BP and subsequent CVD risk than either sodium or potassium alone [12,14,15]. Although there is no generally accepted guideline for the dietary Na/K ratio, an international cooperative study on electrolytes and other factors associated with BP (the INTERSALT study) recommended a urinary molar Na/K ratio of 1.0 as the target level [16], and the World Health Organization (WHO) suggested that following the WHO guidelines on sodium and potassium intakes would result in a molar Na/K ratio of approximately 1.0 [17].

In contrast to the well-established relationship between sodium and potassium intakes and BP and CVD, the one between sodium and potassium intakes and other cardiometabolic risk factors is more controversial. While a high sodium intake has been linked to obesity, type 2 diabetes, and MetS [18,19,20], the association between dietary potassium and these conditions is inconsistent, although a low serum potassium level is reported as an independent predictor of diabetes [20,21]. Furthermore, there is limited research on the association between cardiometabolic risk factors and the dietary Na/K ratio, particularly at a population-based level.

Although 24 h urinary excretion of sodium and potassium has been considered the gold standard for estimating sodium and potassium intakes [22], it is time-consuming and onerous to collect 24 h urine. As a result, dietary recall and record methods are often used to estimate sodium and potassium intakes in large population studies. The Korean National Health and Examination Survey (KNHANES) has included a 24 h diet recall for many years, providing dietary information that cannot be obtained through urinary collection, such as food and nutrient intake. Given the importance of national health surveys in establishing a public health policy, it is essential to investigate the association between the Na/K ratio, based on 24 h diet recall data, and cardiometabolic risk factors. This study aimed to examine this association and identify the food groups related to a higher or lower dietary Na/K ratio in Korean adults, using KNHANES results.

## 2. Materials and Methods

### 2.1. Study Design and Participants

This study analyzed data from KNHANES VII (2016–2018). KNHANES is a continuous, cross-sectional survey with nationally representative Korean citizens selected by a stratified and multi-stage clustered probability design. The survey consists of a health interview, a health examination, and a dietary survey. A detailed description of the survey procedures has been reported elsewhere [23]. Because KNHANES is conducted by the Korean government for public welfare, it has been exempted from deliberation by the bioethics law since 2015. All participants signed their informed consent for inclusion. Among the adults aged 30 years or above who participated in a 24 h dietary recall survey, those with missing biochemical or anthropometric measurements, were pregnant or lactating, or reported implausible energy intake (<500 or >5000 kcal/day) were excluded. A total of 12,996 participants were included in the final analysis.

### 2.2. Collection of Demographic, Anthropometric, and Biochemical Information

Sociodemographic information obtained from the health interview included sex, age, household income, education, and lifestyle factors, such as alcohol consumption, current smoking status, and physical activity. Alcohol consumption was defined as “yes” if the participants drank alcoholic beverages more than once a month during the past year. Participants were regarded as current smokers if they had smoked ≥100 cigarettes in their lifetime and continued smoking. Physical activity was defined as “yes” if participants reported ≥30 min walking more than five times a week. Anthropometric and biochemical assessment data were obtained from the health examination. This study used the data for height, weight, and cardiometabolic risk biomarkers, including systolic and diastolic blood pressures (SBP and DBP), waist circumference (WC), and the concentrations of fasting glucose, triglycerides (TGs), cholesterol, high-density lipoprotein (HDL) cholesterol, and low-density lipoprotein (LDL) cholesterol in the blood. Body mass index (BMI) was calculated by dividing weight (kg) by height squared (m^2^).

### 2.3. Classification According to Dietary Na/K Ratio

Dietary sodium and potassium intakes were estimated using the one-day 24 h dietary recall data. Molar ratios of dietary Na/K were calculated for each participant as the daily sodium intake divided by the daily potassium intake. In order to examine whether cardiometabolic risk factors are associated with the dietary Na/K ratio, participants were categorized into three groups based on their dietary Na/K ratio. These groups were referred to as the low group (first tertile), middle group (second tertile), and high group (third tertile) in relative terms. In addition, the percentage of participants complying with the WHO-recommended molar ratio of Na/K of ≤1.0 (based on WHO guidelines for sodium and potassium intakes) was calculated.

$$\mathrm{Na}/\mathrm{Kratio}\;\left(\mathrm{mmol}/\mathrm{mmol}\right)=\frac{\mathrm{Daily}\;\mathrm{sodium}\;\mathrm{intake}\;\left(\mathrm{mg}\right)/23\left(\mathrm{molecular}\;\mathrm{weight}\;\mathrm{of}\;\mathrm{sodium}\right)}{\;\mathrm{Daily}\;\mathrm{potassium}\;\mathrm{intake}\;\left(\mathrm{mg}\right)/39\left(\mathrm{molecular}\;\mathrm{weight}\;\mathrm{of}\;\mathrm{potassium}\right)}$$

### 2.4. Definitions of Cardiometabolic Risk Factors

In this study, cardiometabolic risk factors included hypertension, obesity, diabetes, and MetS. Hypertension was defined as a SBP ≥ 140 mmHg, DBP ≥ 90 mmHg, or the use of antihypertensive medication [24]. Obesity was defined as BMI ≥ 25 kg/m^2^, according to the WHO Asia–Pacific guidelines [25]. The diagnostic criteria of diabetes were fasting blood glucose ≥ 126 mg/dL, taking oral antidiabetic medications or insulin, or a physician’s diagnosis [26]. MetS was defined according to the criteria established by the American Heart Association (AHA)/National Heart, Lung, and Blood Institute (NHLBI) [5], with the adoption of the WC cutoff value for central obesity in Koreans [27]. The MetS was diagnosed when a study participant presented with any three or more of the following: (1) abdominal obesity (WC ≥ 90 cm for men and ≥85 cm for women), (2) elevated TGs (≥150 mg/dL), (3) low HDL cholesterol (<40 mg/dL for men and <50 mg/dL for women), (4) elevated BP (SBP ≥ 130 mmHg, DBP ≥ 85 mmHg, or taking antihypertensive medications), and (5) elevated fasting blood glucose (≥100 mg/dL) or taking oral antidiabetic medications or insulin.

### 2.5. Assessment of Dietary Factors

Daily calorie and food intakes were estimated using the KNAHNES data on the one-day 24 h dietary recall collected by trained nutritionists. Foods were categorized into 18 food groups based on the food classification of the Korean Rural Development Administration’s food composition table [28]. The cereals + grains group was subdivided into whole grains + rice cakes, breads + cookies, breakfast cereals, and noodles. The vegetable group was divided into salted (such as kimchi and pickled vegetables) and unsalted (all vegetables except kimchi and pickled vegetables) vegetables, and the meat group was classified into three categories: non-marinated meat, meat dishes, and cured and processed meat. To identify the food groups associated with a lower dietary Na/K ratio in Korean adults, the daily intakes of food groups were analyzed by the tertile of the Na/K ratio.

### 2.6. Statistical Analysis

All statistical analyses were performed using the SAS software package (version 9.4; SAS Institute, Cary, NC, USA). The PROC SURVEY procedure in SAS was applied to incorporate the complex sampling design parameters of KNHANES, including strata, cluster, and weight. The general characteristics data are presented as numbers and percentages for categories and as means with standard deviations (SD) for numerical values. Differences in participants’ general characteristics between the groups were compared using the chi-square test for categorical variables and analysis of variance (ANOVA), followed by Tukey’s test for numerical variables. The associations between dietary Na/K ratio and cardiometabolic biomarkers were assessed by Pearson correlation analysis. Means of daily food group intakes were compared by analysis of covariance (ANCOVA) according to the tertile groups. Multiple logistic regression was used to determine odds ratios (ORs) and 95% confidence intervals (CIs) for cardiometabolic risk factors according to the dietary Na/K ratio. To address confounding factors, a stepwise selection method was employed in the multiple regression analysis. This approach involved including potential variables in the regression model and assessing their impact on the regression coefficients and significance levels to identify confounding variables. The final regression model was adjusted for confounding factors such as age, sex, household income, education level, alcohol consumption, current smoking status, physical activity, household composition, economic activity status, and daily energy intake. Participants in the first tertile of the dietary Na/K ratio were considered the reference group for OR and 95% CI estimation. Additionally, to account for variations in health risk across different life stages, we performed an age-stratified analysis to investigate cardiometabolic biomarkers and risk factors according to the dietary Na/K ratio. Participants were categorized into three age groups: 30–49 years, representing the onset of cardiometabolic disease [29]; 50–65 years, exhibiting a notable surge in cardiometabolic disease prevalence [29]; 65 years or above, commonly used to define the senior citizen population. Tests for trend were performed to examine linearities between the tertiles of the dietary Na/K ratio and cardiometabolic risk and daily intakes of each food group. A *p*-value < 0.05 was considered statistically different.

## 3. Results

### 3.1. Dietary Sodium and Potassium Intakes and General Characteristics of Study Population

Table 1 presents the mean sodium and potassium daily intakes and molar Na/K ratio in Korean adults aged 30 years and above. The average daily intakes of sodium and potassium in all participants were 3324 and 2867 mg, respectively. The mean dietary molar Na/K ratio in all participants was 2.12, and when divided into thirds, the mean molar ratios were 1.11 (low), 1.92 (medium), and 3.21 (high). When assessing compliance with the target molar Na/K ratio, only 11.4% of the participants had a molar ratio of Na/K ≤ 1.00. In the low, medium, and high Na/K ratio groups, the mean daily intakes of sodium were 2039, 3239, and 4694 mg, and those of potassium were 3188, 2870, and 2542 mg, respectively.

The characteristics of the participants are summarized in Table 2. When classified into thirds according to the dietary Na/K ratio, significant associations were found between the general characteristics of the participants and the level of the Na/K ratio. The high Na/K ratio group was more likely to be younger and have more men, a higher BMI, less alcohol consumption, less physical activity, and more current smokers than the low Na/K ratio group. In addition, total daily energy intake was high in the high Na/K ratio group. Thus, the subsequent analyses of the associations of the Na/K ratio level with cardiometabolic risk factors and food group intakes were adjusted by sociodemographic variables and lifestyle factors.

### 3.2. Distribution of Cardiometabolic Risk Factors and Biomarkers

**Figure 1:**
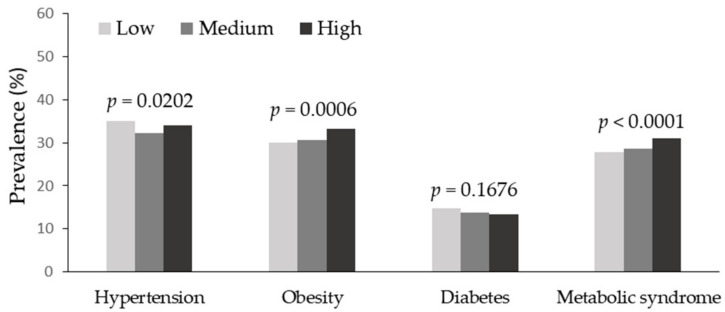
Prevalence of cardiometabolic risk factors by the tertile of sodium-to-potassium intake ratio.

Figure 1 shows the prevalence of cardiometabolic risk factors. The prevalence of hypertension, obesity, diabetes, and MetS in all participants was 33.7%, 31.4%, 13.9%, and 29.1%, respectively. There were significant differences in the prevalence of hypertension (*p* = 0.0202), obesity (*p* = 0.0006), and MetS (*p* < 0.0001) according to the dietary Na/K ratio level. Unlike the nonlinear dose–response relation between the dietary Na/K ratio level and hypertension, the prevalence of obesity and MetS increased as the dietary Na/K ratio increased.

The biomarkers related to cardiometabolic risk factors are presented in Table 3. Significant associations were observed between the dietary Na/K ratio and DBP, serum TGs, HDL cholesterol, and WC in all participants. The high Na/K ratio group exhibited higher DBP, serum TGs, and WC but lower HDL cholesterol compared to the low Na/K ratio group. Conversely, there was no significant association between the dietary Na/K ratio and SBP or fasting blood glucose in all participants. However, when stratified by age, a significant positive association between SBP and dietary Na/K ratio was found among participants aged 30–49 years and those aged 65 years or above. Similarly, a similar relationship was observed between fasting blood glucose and the dietary Na/K ratio in adults aged 30–49 years and 50–64 years.

### 3.3. Association of Dietary Na/K Ratio with Cardiometabolic Risk Factors

We performed multiple logistic regression analyses to investigate the associations of cardiometabolic risk factors with the level of the Na/K ratio. Table 4 shows the ORs and 95% CIs for these risk factors based on the Na/K ratio level after adjusting for confounding variables. Our result showed no significant association between the dietary Na/K ratio and hypertension, obesity, and diabetes in all participants. However, a high Na/K ratio was linked to an increased risk of MetS compared to a low Na/K ratio (OR = 1.22, 95% CI: 1.08–1.38). Additionally, a positive, linear dose–response relationship was found between the dietary Na/K ratio and the risk of MetS (*p* for trend = 0.0020). When stratified by age, we observed different responses for hypertension and MetS according to the Na/K ratio level. While a higher Na/K ratio was not linked to a higher hypertension risk in younger participants, it was associated with a higher risk of hypertension in older adults aged 65 years or above (OR = 1.38, 95% CI: 1.10–1.72). Furthermore, the dietary Na/K ratio was positively associated with hypertension risk in older adults (*p* for trend = 0.0159). In terms of MetS, a positive association with the dietary Na/K ratio was found in participants aged 30–49 years (*p* for trend = 0.0211) and, likewise, in those aged 65 years or above (*p* for trend = 0.0044). However, only the high Na/K ratio was associated with a higher MetS risk compared to the low Na/K ratio in adults aged 30–49 years (OR = 1.29, 95% CI: 1.05–1.60). In older adults aged 65 years or above, both the medium and high Na/K ratio groups showed higher ORs for MetS (ORs [95% CIs] = 1.31 [1.09–1.56] and 1.28 [1.06–1.54], respectively) compared to the low Na/K ratio group.

In addition, we further analyzed the associations of the dietary Na/K ratio with the five components of MetS. Compared to the low Na/K ratio, the high Na/K ratio was associated with an increased risk of abdominal obesity (OR = 1.16, 95% CI: 1.03–1.30), elevated serum TGs (OR = 1.20, 95% CI: 1.07–1.34), and elevated BP (OR = 1.12, 95% CI: 1.01–1.25) in all participants. The middle Na/K ratio group also showed a higher OR for elevated BP (OR = 1.12, 95% CI: 1.01–1.25). Positive linear trends were found between the Na/K ratio levels and these MetS components (*p* for trend = 0.0238 for abdominal obesity, *p* for trend = 0.0008 for elevated serum TGs, and *p* for trend = 0.0430 for elevated BP). When we analyzed the data according to age groups, responses differed according to the age groups. In adults aged 30–49, the response to dietary Na/K level was almost the same as that in all participants. The high Na/K ratio group had a higher risk of abdominal obesity (OR = 1.25, 95% CI: 1.02–1.53), elevated TGs (OR = 1.30, 95% CI: 1.06–1.58), and elevated BP (OR = 1.25, 95% CI: 1.02–1.53) compared to the low Na/K ratio group. The middle Na/K ratio group also showed a higher OR for elevated BP (OR = 1.24, 95% CI: 1.00–1.55). Positive correlations were found between the dietary Na/K ratio and the risks of abdominal obesity (*p* for trend = 0.0383) and elevated TGs (*p* for trend = 0.0041). In adults aged 50–64 years, only the risk of elevated serum TGs was increased by the high Na/K ratio (OR = 1.26, 95% CI: 1.03–1.53), with a positive association according to the Na/K ratio level (*p* for trend = 0.0253). In older adults aged 65 or above, BP risk was increased in both the medium (OR = 1.24, 95% CI: 1.04–1.48) and high (OR = 1.27, 95% CI: 1.04–1.55) Na/K ratio groups, with a positive linear association (*p* for trend = 0.0190) compared to the low Na/K ratio group. The OR for abdominal obesity was higher in the medium Na/K ratio group (OR = 1.24; 95% CI: 1.03–1.50) compared to the low Na/K ratio group.

### 3.4. Association of Dietary Na/K Ratio with Intakes of Food Groups

Table 5 presents the daily intakes of food groups across the tertile of the dietary Na/K ratio. Food groups positively associated with a lower Na/K ratio were potato + potato products, nuts, fruits, and dairy products (all *p* < 0.001). Food groups negatively associated with a lower Na/K ratio were cereals + grains, vegetables, meat + meat dishes, eggs, fish, oils, seasoning, and processed foods (*p* < 0.001 except vegetables with *p* = 0.0021). When cereals + grains were separated into subgroups, intakes of breads + cookies and noodles showed an inverse linear trend with a lower Na/K ratio (*p* for trend = 0.0346 for breads + cookies, and *p* for trend = 0.0001 for noodles), but without an association between the Na/K ratio and whole grains + rice cakes, which constitute the majority of cereals + grains. The vegetable group also shows different associations when divided into salted and unsalted vegetables. Salted vegetable intake was negatively associated with a lower Na/K ratio, whereas unsalted vegetable intake was positively associated with a lower Na/K ratio (*p* for trend = 0.0001).

## 4. Discussion

The present study estimated the dietary Na/K ratio based on 24 h diet recall and investigated the association between the Na/K ratio and cardiometabolic risk factors in nationally representative Korean adults aged 30 years and above. The mean sodium intake for all participants was 3324 mg/day, a value much higher than the WHO guideline (<2000 mg/day) and the Korean CDRR of 2300 mg/day for sodium. In contrast, the mean potassium intake of 2869 mg/day was considerably below the WHO guideline (>3510 mg/day) and the Korean AI of 3500 mg/day for potassium. High-sodium and low-potassium intakes resulted in a mean molar Na/K ratio of 2.08, with 88.6% of Korean adults exceeding the target molar Na/K ratio of ≤1.00. A recent study obtained a similar mean dietary molar Na/K ratio of 2.33 in Korean adults [30]. In that study, the mean sodium and potassium intakes and molar Na/K ratio based on 24 h diet recall were comparable to those measured through 24 h urine collection, with differences of −9.4%, −2.7%, and −6.8%, respectively. This finding is consistent with a National Health and Nutrition Examination Survey (NHANES) of the overall United States population that sodium intake estimation by 24 h dietary recall had an approximate 9% underestimation compared to 24 h urinary collection [31]. These studies suggest that 24 h dietary recall is acceptable to estimate dietary intake at the population level.

Several studies have shown that the Na/K ratio is associated with BP and hypertension [14,15,16]. However, in this study, the dietary Na/K ratio did not have a positive linear association with the prevalence of hypertension and SBP. This could be attributed to the inclusion of participants with self-reported physician-diagnosed hypertension, stroke, or coronary heart diseases in the study. These individuals may have changed their diets after diagnosis with these diseases, leading to the high prevalence of hypertension in the low Na/K ratio group observed in our result. Furthermore, multivariate logistic regression analysis did not find a significant association between the Na/K ratio and the risk of hypertension in all participants. However, older adults (≥65 years) with a high Na/K ratio were found to have a higher risk of hypertension compared to those with a low Na/K ratio. Along with the increase in SBP with age, it may be because the BP of older adults is more sensitive to salt intake compared to younger adults [32]. A longitudinal study in the Japanese general population reported that the positive association between the Na/K ratio and SBP was steeper in older adults [33]. The increased salt sensitivity in older adults is not necessarily due to increased salt intake but rather to a decreased ability to excrete sodium. This reduced capacity for sodium excretion in older adults is due to several factors, including a decline in renal function, reduced synthesis of natriuretic substances, and reduced activity of membrane sodium/potassium-adenosine triphosphatase (Na/K-ATPase) [32,34].

This study shows that a higher Na/K ratio is linked to an increase in BMI and prevalence of obesity, and the Na/K ratio was positively associated with WC. A previous study suggested that the association between a high sodium intake and body weight gain is possibly due to the high energy intake because a high salt intake is associated with increased thirst and ingestion of sweetened soft drinks [35]. Similarly, our study observed a significantly high daily energy intake in participants with a high Na/K ratio. However, after adjusting for confounding factors, including total energy intake, we found that the dietary Na/K ratio was not significantly associated with the risk of obesity defined by BMI. Instead, it was positively associated with the risk of abdominal obesity assessed by WC. This finding suggests that the Na/K ratio is an independent predictor of abdominal obesity risk. To date, only a few studies have examined the association between the Na/K ratio and the risk of obesity. A cross-sectional study in Japan showed that spot urine Na/K ratio was positively associated with BMI, independently of confounding factors [14]. In a study in China, a higher Na/K ratio was significantly linked to an increased risk of abdominal obesity but not overweight and obesity defined by BMI, whereas 24 h urinary Na excretion was positively associated with both general obesity and abdominal obesity [36]. Furthermore, a multiethnic cohort study showed that the spot urine Na/K ratio was independently associated with total body fat measured by dual-energy X-ray absorptiometry [37]. Therefore, these findings collectively suggest that a high Na/K ratio could increase the risk of obesity, especially abdominal obesity.

There has been little research on the impact of the Na/K ratio on MetS, even though a high Na/K ratio can affect more than just BP by increasing the risk of obesity. Our study found a positive association between the dietary Na/K ratio and the risk of MetS. Among the components of MetS, the risk of elevated serum TGs and elevated BP was positively associated with the Na/K ratio level, in addition to abdominal obesity, as aforementioned. Supporting our findings, a previous study in China reported a positive association between the 24 h urinary Na/K ratio and the risk of MetS, increasing the odds of abdominal obesity and elevated BP, although not affecting the odds of elevated serum TGs, low HDL cholesterol, and elevated fasting glucose [38]. Several studies have identified a high Na intake as a risk factor for MetS [20,39,40]. Meanwhile, studies examining the association between potassium intake and MetS are limited and inconsistent. One study on Chinese adults found no relationship between potassium excretion and MetS [20]. However, a meta-analysis based on cross-sectional studies demonstrated an inverse association between MetS prevalence and potassium intake [21]. Furthermore, a recent survey in Israel showed that potassium excretion and the Na/K excretion ratio were correlated with MetS, and both were associated with obesity and fatty liver [41]. Considering that the accumulation of visceral fat activates the production of pro-inflammatory cytokines and delivers excess amounts of free fatty acids to the liver via the portal vein, resulting in the synthesis of very low-density lipoprotein [42], abdominal obesity emerges as a predominant risk factor for MetS, contributing to hyperlipidemia, hypertension, and insulin resistance.

We determined the food groups associated with a lower dietary Na/K ratio in Korean adults, an essential step that can aid in developing strategies to modify the population’s dietary intake. The food groups that showed a positive association with a lower Na/K ratio were potatoes, fruits, nuts, and dairy products. In contrast, cereal + grains, vegetables, meat, eggs, fish, oils, seasoning, and processed foods exhibited a negative association with a lower Na/K ratio. Overall intake of fruits and vegetables is considered an indicator of a healthy diet to reduce non-communicable diseases like CVD and cancer [43]. Moreover, high consumption of fruits and vegetables has been reported to be associated with a lower risk of MetS [44]. In our study, we found a positive association between high fruit intake and a lower Na/K ratio, whereas a low vegetable intake was linked to a lower Na/K ratio. When we divided vegetables into salted and unsalted categories, we discovered that the higher vegetable intake in the high Na/K ratio group was primarily due to the high consumption of salted vegetables, such as kimchi, a major traditional food in Korea. However, unsalted vegetable intake was positively associated with a lower Na/K ratio. This finding aligns with previous reports that vegetable intake was less effective in decreasing cardiometabolic risk compared to fruit intake in the Korean population [45,46]. Similarly, we observed that the intake of processed foods and seasoned dishes was higher in the cereals + grains and meat groups among individuals with a higher Na/K ratio. Therefore, in addition to increasing the consumption of food groups positively associated with a lower Na/K ratio, efforts should be made to choose less salty processed food to reduce the Na/K ratio.

This study has some limitations. First, the cross-sectional study design hindered the inference of direct causality between the Na/K ratio and cardiometabolic risk factors. Second, one-day 24 h dietary recall data may not be enough to estimate usual daily sodium and potassium intakes and their ratio because multiple 24 h recalls are the preferred valid method for estimating intakes [47]. Lastly, this study could not consider hidden variables that might affect the association between the Na/K ratio and cardiometabolic risk factors, such as other dietary factors, drugs, or nutritional supplements. Despite these limitations, this is the first nationally representative population-based study to investigate associations between cardiometabolic risk factors and the dietary Na/K ratio. Although our results showed an independent inverse association between the dietary Na/K ratio and the risk of MetS and its components, further studies are needed to establish the causal relationship between the Na/K ratio and MetS and the long-term outcome of MetS according to the Na/K ratio level.

## 5. Conclusions

This study showed that the estimated mean dietary Na/K ratio in Korean adults was 2.08, and a higher Na/K ratio was associated with an increased risk of hypertension in older adults and an increased risk of MetS in all participants. Among the risk factors of MetS, abdominal obesity, elevated TGs, and elevated BP showed positive associations with the Na/K ratio. The food groups that exhibited a positive association with a lower Na/K ratio were fruits, unsalted vegetables, nuts, potatoes, and dairy products. Therefore, public health strategies should prioritize efforts to reduce sodium intake and increase potassium consumption in the usual diet in order to reduce MetS prevalence and subsequent cardiometabolic diseases.

## Figures and Tables

**Table 1:** Sodium and potassium intakes, Na/K ratio, and compliance with the target ratio according to the tertile of dietary Na/K ratio.

	Total (*n* = 12,996)	Low (*n* = 4332)	Medium (*n* = 4332)	High (*n* = 4332)	*p*-Value
Sodium (mg)	3324 ± 2127	2039 ± 1124 ^c^	3239 ± 1473 ^b^	4694 ± 2569 ^a^	<0.0001
Potassium (mg)	2867 ± 1413	3188 ± 1638 ^a^	2870 ± 1270 ^b^	2542 ± 1218 ^c^	<0.0001
Na/K (mmol/mmol)	2.08 ± 0.10	1.11 ± 0.32 ^c^	1.92 ± 0.21 ^b^	3.21 ± 1.09 ^a^	<0.0001
Na/K ≤ 1.0 * (%)	1476 (11.4)	1476 (34.1)	0 (0)	0 (0)	<0.0001

Values are presented as mean ± SD or *n* (%). * WHO recommendation (molar Na/K ≤ 1.00). Mean values without the same superscripts (a, b, and c) are significantly different between dietary Na/K ratio groups by ANOVA, followed by Tukey’s test.

**Table 2:** General characteristics of participants according to tertile of dietary sodium-to-potassium ratio.

Characteristics		Low (*n* = 4332)	Medium (*n* = 4332)	High (*n* = 4332)	*p*-Value
Sex	Men	1365 (31.5)	1894 (43.7)	2155 (49.8)	<0.0001
Age group (years)	30–49	1234 (28.5)	1796 (41.5)	2024 (46.7)	
	50–64	1586 (36.6)	1345 (31.0)	1213 (28.0)	<0.0001
	≥65	1512 (34.9)	1191 (27.5)	1095 (25.3)	
Body mass index (kg/m^2^)		23.89 ± 3.31 ^b^	24.01 ± 3.37 ^b^	24.25 ± 3.60 ^a^	<0.001
Household income	Lowest	925 (21.4)	784 (18.1)	908 (21.0)	
	Lower-middle	1066 (24.6)	1063 (24.5)	1067 (24.6)	<0.0001
	Upper-middle	1084 (25.0)	1201 (27.7)	1214 (28.0)	
	Highest	1257 (29.0)	1284 (29.6)	1143 (26.4)	
Education	Middle school or less	1702 (39.3)	1407 (32.5)	1412 (32.7)	
	High school	1234 (28.5)	1211 (28.0)	1309 (30.2)	<0.0001
	College or above	1396 (32.2)	1714 (39.6)	1611 (37.2)	
Alcohol consumption	Yes	2524 (58.4)	1982 (45.8)	1785 (41.3)	<0.0001
Current smoking status	Yes	443 (10.2)	710 (16.4)	969 (21.7)	<0.0001
Physical activity	Yes	1707 (39.5)	1577 (36.5)	1448 (33.5)	<0.0001
Daily energy intake (kcal)		1791 ± 791 ^c^	1966 ± 846 ^b^	2009 ± 928 ^a^	<0.001

Values are presented as *n* (%) or mean ± SD. Alcohol consumption: “yes” consumed alcoholic beverages more than once a month during the past year. Current smoking status: “yes” smoked ≥ 100 cigarettes in a lifetime and a current smoker. Physical activity: “yes” performed ≥ 30 min walking more than five times a week. The chi-square test and ANOVA analysis followed by Tukey’s test were used for categorical variables and continuous variables, respectively. Mean values without the same superscripts (a, b, and c) are significantly different between dietary Na/K ratio groups by ANOVA, followed by Tukey’s test.

**Table 3:** Cardiometabolic risk biomarkers according to the tertile of dietary sodium-to-potassium ratio by age.

Cardiometabolic Risk Biomarkers	Na/K Ratio Tertile	Total (*n* = 12,996)	Age 30–49 Years (*n* = 5054)	Age 50–64 Years (*n* = 4144)	Age ≥ 65 Years (*n* = 3798)
Systolic blood pressure (mmHg)	Low	120.4 ± 17.1	111.2 ± 13.5 ^c^	120.5 ± 16.3	127.9 ± 17.1
	Medium	119.7 ± 17.2	112.4 ± 13.5 ^b^	121.7 ± 16.3	128.7 ± 17.5
	High	119.9 ± 16.8	113.7 ± 13.6 ^a^	121.7 ± 16.3	129.6 ± 17.6
	Correlation ^1^	−0.013	0.075 ***	0.026	0.042 *
Diastolic blood pressure (mmHg)	Low	74.9 ± 9.9 ^b^	75.0 ± 9.9 ^c^	77.3 ± 9.2 ^b^	72.1 ± 9.7
	Medium	75.9 ± 10.2 ^a^	76.3 ± 10.4 ^b^	78.5 ± 9.6 ^a^	72.9 ± 10.1
	High	76.3 ± 10.5 ^a^	77.4 ± 10.6 ^a^	78.5 ± 9.9 ^a^	72.1 ± 10.0
	Correlation	0.054 ***	0.087 ***	0.049 **	−0.007
Fasting blood glucose (mg/dL)	Low	102.3 ± 23.8	95.7 ± 19.7 ^b^	102.6 ± 23.4 ^b^	107.2 ± 26.1
	Medium	102.5 ± 25.2	96.7 ± 20.5 ^ab^	104.2 ± 24.7 ^ab^	108.9 ± 27.8
	High	103.0 ± 26.2	98.3 ± 21.7 ^a^	106.3 ± 30.2 ^a^	108.8 ± 28.1
	Correlation	0.010	0.038 **	0.059 ***	0.022
Serum triglycerides (mg/dL)	Low	129.8 ± 93.1 ^c^	125.1 ± 119.5 ^b^	130.5 ± 88.4 ^c^	131.9 ± 75.3
	Medium	137.0 ± 111.1 ^b^	133.9 ± 122.4 ^b^	142.7 ± 106.2 ^b^	134.9 ± 77.5
	High	148.9 ± 128.2 ^a^	149.3 ± 128.9 ^a^	162.3 ± 149.9 ^a^	137.3 ± 97.6
	Correlation	0.070 ***	0.084 ***	0.095 ***	0.024
Correlation	Low	193.5 ± 37.9	194.8 ± 34.5	200.5 ± 38.5	186.4 ± 38.7
	Medium	193.9 ± 38.8	195.8 ± 34.8	199.3 ± 38.8	184.0 ± 40.1
	High	193.5 ± 37.9	197.0 ± 34.8	197.9 ± 40.0	183.1 ± 40.9
	Correlation	0.001	0.032 *	−0.036 *	−0.023
Serum HDL cholesterol (mg/dL)	Low	51.2 ± 12.7 ^a^	54.0 ± 13.2 ^a^	51.9 ± 12.5 ^a^	48.8 ± 11.5 ^a^
	Medium	50.8 ± 12.8 ^ab^	52.3 ± 12.7 ^b^	50.9 ± 13.2 ^ab^	47.5 ± 12.1 ^b^
	High	50.3 ± 12.5 ^b^	51.8 ± 12.7 ^b^	50.0 ± 12.5 ^b^	47.9 ± 11.9 ^ab^
	Correlation	−0.029 **	−0.068 ***	−0.054 ***	−0.017
Serum LDL cholesterol (mg/dL)	Low	116.5 ± 34.4	117.6 ± 33.5	121.4 ± 32.8 ^a^	107.2 ± 35.0
	Medium	117.0 ± 36.5	122.2 ± 34.2	117.9 ± 35.6 ^ab^	110.3 ± 38.5
	High	115.9 ± 37.2	123.6 ± 34.5	111.0 ± 38.1 ^b^	109.0 ± 39.3
	Correlation	−0.007	0.041	−0.117 **	−0.015
Waist circumference (cm)	Low	82.1 ± 9.6 ^c^	79.3 ± 10.3 ^c^	81.9 ± 9.0 ^c^	84.9 ± 9.0 ^b^
	Medium	82.9 ± 9.8 ^b^	81.0 ± 10.4 ^b^	83.1 ± 8.8 ^b^	85.7 ± 8.8 ^a^
	High	83.7 ± 10.1 ^a^	82.7 ± 10.7 ^a^	84.2 ± 9.4 ^a^	85.6 ± 9.1 ^a^
	Correlation	0.066 ***	0.126 ***	0.100 ***	0.042 **

Values are presented as mean ± SD. Mean values without the same superscripts (a, b, and c) are significantly different between dietary Na/K ratio groups within age groups (column) by ANOVA, followed by Tukey’s test (*p* < 0.05). ^1^ Correlation coefficient by Pearson correlation analysis (* *p* < 0.05, ** *p* < 0.01, *** *p* < 0.001).

**Table 4:** Multivariable-adjusted odds ratios (95% CI) for cardiometabolic risk factors according to the tertile of dietary sodium-to-potassium ratio by age.

Risk Factors	Na/K Ratio Tertile	Total (*n* = 12,996)	Age 30–49 Years (*n* = 5054)	Age 50–64 Years (*n* = 4144)	Age ≥ 65 Years (*n* = 3798)
Hypertension	Low	Reference	Reference	Reference	Reference
	Medium	0.99 (0.85–1.14)	0.71 (0.46–1.09)	0.99 (0.78–1.24)	1.17 (0.95–1.44)
	High	1.07 (0.92–1.24)	0.80 (0.53–1.19)	0.98 (0.78–1.24)	1.38 (1.10–1.72)
	*p* for trend	0.5370	0.3563	0.9849	0.0159
Obesity	Low	Reference	Reference	Reference	Reference
	Medium	0.98 (0.89–1.09)	0.99 (0.82–1.19)	1.00 (0.84–1.19)	1.07 (0.88–1.29)
	High	1.05 (0.94–1.17)	1.10 (0.92–1.33)	0.99 (0.82–1.21)	1.09 (0.91–1.32)
	*p* for trend	0.4183	0.3373	0.9987	0.6199
Diabetes	Low	Reference	Reference	Reference	Reference
	Medium	1.04 (0.91–1.20)	0.98 (0.63–1.51)	1.09 (0.85–1.39)	1.06 (0.88–1.27)
	High	1.07 (0.92–1.24)	1.05 (0.68–1.60)	1.21 (0.93–1.57)	0.94 (0.76–1.16)
	*p* for trend	0.5800	0.8907	0.3654	0.5702
Metabolic syndrome	Low	Reference	Reference	Reference	Reference
	Medium	1.05 (0.93–1.18)	1.05 (0.85–1.30)	0.96 (0.79–1.16)	1.31 (1.09–1.56)
	High	1.22 (1.08–1.38)	1.29 (1.05–1.60)	1.13 (0.93–1.38)	1.28 (1.06–1.54)
	*p* for trend	0.0020	0.0211	0.2729	0.0044
Components of metabolic syndrome					
Abdominal obesity	Low	Reference	Reference	Reference	Reference
	Medium	1.02 (0.92–1.14)	1.05 (0.86–1.29)	0.93 (0.77–1.12)	1.24 (1.03–1.50)
	High	1.16 (1.03–1.30)	1.25 (1.02–1.53)	1.05 (0.86–1.28)	1.20 (0.99–1.45)
	*p* for trend	0.0238	0.0383	0.4648	0.0560
Elevated triglyceride	Low	Reference	Reference	Reference	Reference
	Medium	0.99 (0.89–1.11)	1.02 (0.83–1.26)	0.98 (0.81–1.18)	1.11 (0.93–1.34)
	High	1.20 (1.07–1.34)	1.30 (1.06–1.58)	1.26 (1.03–1.53)	0.99 (0.81–1.21)
	*p* for trend	0.0008	0.0041	0.0253	0.4233
Reduced HDL cholesterol	Low	Reference	Reference	Reference	Reference
	Medium	0.96 (0.86–1.07)	0.91 (0.76–1.09)	0.91 (0.75–1.09)	1.17 (0.98–1.40)
	High	0.99 (0.88–1.10)	0.97 (0.81–1.16)	0.98 (0.81–1.19)	1.01 (0.83–1.21)
	*p* for trend	0.6952	0.5254	0.5211	0.1672
Elevated blood pressure	Low	Reference	Reference	Reference	Reference
	Medium	1.12 (1.01–1.25)	1.24 (1.00–1.55)	1.04 (0.86–1.27)	1.24 (1.04–1.48)
	High	1.12 (1.01–1.25)	1.25 (1.02–1.53)	0.97 (0.80–1.17)	1.27 (1.04–1.55)
	*p* for trend	0.0430	0.0546	0.7791	0.0190
Elevated fasting blood glucose	Low	Reference	Reference	Reference	Reference
	Medium	1.02 (0.91–1.14)	0.94 (0.77–1.15)	1.12 (0.93–1.35)	1.04 (0.87–1.24)
	High	1.10 (0.99–1.23)	1.07 (0.87–1.31)	1.16 (0.96–1.40)	1.09 (0.91–1.32)
	*p* for trend	0.1593	0.3631	0.2938	0.6383

Values are presented as odds ratios (95% CI). Multiple logistic regression analysis, adjusted for age, sex, household income, education level, alcohol consumption, current smoking status, physical activity, household composition, economic activity status, and total energy intake. Metabolic syndrome was defined as ≥3 components of the following: (1) abdominal obesity (waist circumference ≥90 cm for men, ≥85 cm for women); (2) hypertriglyceridemia (≥150 mg/dL); (3) low HDL (<40 mg/dL for men, <50 mg/dL for women); (4) elevated blood pressure (≥130/85 mmHg); (5) elevated fasting blood glucose (≥100 mg/dL). Abbreviation: CI, Confidence Intervals.

**Table 5:** Daily intakes of food groups according to the tertile of dietary sodium-to-potassium ratio.

Intakes by Food Groups (g/Day)	Low (*n* = 4332)	Medium (*n* = 4332)	High (*n* = 4332)	*p* for Trend
Cereal + grains	238.5 ± 3.1	282.0 ± 2.8	305.2 ± 3.2	0.0001
Whole grain + rice cakes	201.1 ± 2.6	215.2 ± 2.5	201.7 ± 2.6	0.3725
Breads + cookies	23.1 ± 1.4	27.3 ± 1.2	29.6 ± 1.2	0.0346
Breakfast cereals	0.6 ± 0.1	0.8 ± 0.1	0.4 ± 0.1	0.0263
Noodles	12.3 ± 0.8	34.9 ± 1.6	62.5 ± 2.2	0.0001
Potato + potato products	65.6 ± 3.3	30.2 ± 1.2	18.1 ± 0.7	0.0001
Sugar	6.8 ± 0.3	7.7 ± 0.3	7.9 ± 0.3	0.3916
Beans	38.8 ± 1.4	40.2 ± 1.4	34.5 ± 1.4	0.0651
Nuts	10.0 ± 0.7	7.0 ± 0.4	6.1 ± 0.5	0.0003
Vegetable	302.8 ± 4.6	330.1 ± 3.8	332.9 ± 4.0	0.0021
Salted vegetable	80.5 ± 1.6	124.8 ± 2.0	148.4 ± 2.4	0.0001
Unsalted vegetable	222.3 ± 4.2	205.2 ± 3.1	184.5 ± 2.9	0.0001
Mushroom	6.3 ± 0.4	6.8 ± 0.4	5.8 ± 0.4	0.1454
Fruits	320.7 ± 7.1	184.9 ± 4.6	107.2 ± 3.5	0.0001
Meat + meat dishes	80.1 ± 2.4	118.6 ± 3.0	123.6 ± 3.6	0.0005
Non-marinated meat	53.5 ± 1.8	76.6 ± 2.1	74.0 ± 2.2	0.3047
Meat dishes	22.5 ± 1.4	33.5 ± 2.1	37.3 ± 2.5	0.0136
Cured and processed meat	2.9 ± 1.4	5.9 ± 0.4	7.1 ± 0.5	0.0001
Eggs	25.3 ± 0.8	28.1 ± 0.8	31.8 ± 1.1	0.0001
Fish	82.8 ± 2.4	120.0 ± 3.5	134.8 ± 3.6	0.0001
Seaweed	29.9 ± 1.8	33.4 ± 2.1	27.8 ± 1.6	0.7608
Dairy	107.3 ± 3.3	81.7 ± 2.9	52.1 ± 2.1	0.0001
Milk	71.1 ± 2.7	54.6 ± 2.4	32.4 ± 1.7	0.0001
Yogurt	26.4 ± 1.5	17.2 ± 1.2	10.9 ± 0.8	0.0001
Oils	4.9 ± 0.2	6.6 ± 0.2	7.4 ± 0.2	0.0001
Beverage	252.2 ± 8.6	327.6 ± 9.1	347.1 ± 9.6	0.4729
Seasoning	24.3 ± 0.5	35.5 ± 0.6	44.8 ± 0.8	0.0001
Processed food	8.7 ± 0.8	15.2 ± 1.1	22.6 ± 1.4	0.0001

Values are presented as mean ± SE. *p*-value from ANCOVA, adjusted for age, sex, household income, education level, alcohol consumption, current smoking status, physical activity, household composition, economic activity status, and total energy intake.

## Data Availability

The data presented in this study are available upon request from the corresponding author.
